# Supplementary material for: MetaRibo-Seq measures translation in microbiomes
Source: Nat Commun. 2020 Jun 29;11:3268. doi: 10.1038/s41467-020-17081-z (PMC7324362; doi:10.1038/s41467-020-17081-z)
Supplement: Supplementary file 10 — Supplementary Data 7 [file 41467_2020_17081_MOESM10_ESM.zip › File2/Confidence_High_Taxonomy/157186_out.krona.html]

Javascript must be enabled to view this page.

members
magnitude
magnitudeUnassigned
count
unassigned
taxon
rank

157186\_out


SRS051031\_contig\_number\_35324
1
3

2759
1
superkingdom

kingdom
1
4751

subkingdom
1
451864

4890
phylum
1

147538
1
subphylum

class
1
147541

451867
subclass
1

1
order
134362


SRS013687\_contig\_number\_13895
93133
1
family


SRS098571\_contig\_number\_10402
superkingdom
1
2
